# Left Peripheral Nerve Palsy after Left Anterior Temporal Lobectomy for Mesial Temporal Sclerosis

**DOI:** 10.1055/a-2914-7901

**Published:** 2026-07-23

**Authors:** Luis Andros, Danilo D.C.L. Lemos, Ricardo Silva Centeno

**Affiliations:** 1 Neurosurgery 28105 Universidade Federal de São PauloSão PauloSão PauloBrazil

**Keywords:** anterior temporal lobectomy, drug-resistant epilepsy, facial paralysis, Bell's palsy

## Abstract

We report a rare case of delayed peripheral facial paralysis following left anterior temporal lobectomy (ATL) for mesial temporal sclerosis in a 58-year-old woman with a long-standing history of seizures. Preoperative evaluation confirmed left temporal sclerosis as the epileptogenic focus. Surgery with neurophysiologic monitoring was performed, and initial postoperative assessment revealed no facial deficits. However, 4 days later, the patient developed left-sided peripheral facial paralysis (House–Brackmann grade 4), which improved after a 10-day course of oral corticosteroids. This case underscores that delayed facial paralysis, although uncommon, can occur after ATL, potentially due to mechanical or thermal injury near the geniculate ganglion or viral reactivation induced by direct manipulation or surgical stress. Awareness and timely follow-up are essential for early detection and management of such complications.

## Introduction

Anterior temporal lobectomy (ATL) is the most performed surgical procedure for epilepsy treatment. 1 2 This procedure involves resecting the neocortex and mesial temporal structures, thus working near the temporal cranial base and surrounding structures such as the third cranial nerve, anterior choroidal artery, middle cerebral artery, posterior cerebral artery, and cerebral peduncle. 3

Although delayed peripheral facial nerve palsy following ATL has been described in prior reports and small case series, it remains an uncommon and potentially underrecognized postoperative complication. Its delayed onset may lead to diagnostic uncertainty, delayed treatment, or unnecessary investigations. 4 5 6 Reporting additional cases contributes to reinforcing clinical awareness, refining diagnostic reasoning, and emphasizing the importance of postoperative surveillance and early intervention, particularly in patients without immediate cranial nerve deficits. Furthermore, individual case reports remain valuable in illustrating real-world clinical scenarios, especially when complications are rare, variably reported, and lack standardized diagnostic criteria. Therefore, we present the case of a patient with drug-resistant epilepsy who developed delayed facial paralysis after ATL on postoperative day 4.

## Presentation

We present the case of a 58-year-old female who had her first seizure at the age of 11 years, triggered by physical aggression from her father (an event with loss of consciousness without other details known or remembered by the patient or family).

Since then, she has been using antiseizure medication, and she has never had a significant seizure-free period. She was unable to work for the past 5 years due to epileptic seizures and accidents associated with the events, so she was completely dependent on one of her daughters.

The current semiology of seizures before the surgical procedure was of focal seizure with impaired consciousness. At the beginning of the seizures, she experienced an occasional sensation of tightness in the throat, followed by behavioral arrest, staring, dystonic posture in the upper limbs (with no side preference), and inability to speak, lasting approximately 2 min. She then experienced postictal aggressiveness and drowsiness. All the seizures presented the same semiology, with rare evolution to bilateral tonic–clonic seizures. The current frequency was two to three times per week with the use of adequate doses of topiramate, carbamazepine, and clobazam.

Magnetic resonance imaging of the brain revealed left temporal sclerosis ( Fig. 1 ). The video-electroencephalogram documented five electroclinical seizures beginning in the left anterior and middle temporal region, with two of the five progressing to bilateral tonic-clonic seizures.

**Fig. 1:**
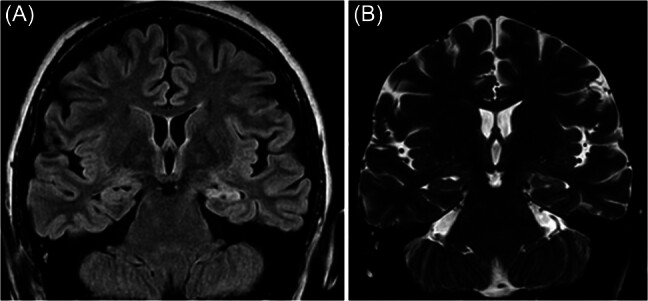
Magnetic resonance imaging of the brain. Coronal T2-FLAIR ( **A** ) and T2 sequences ( **B** ) show hyperintensity and decreased volume of the left hippocampus.

Based on the findings, it was decided to perform a left ATL under neurophysiologic monitoring.

## Surgical Procedure

The patient was placed supine, with a roll under her left side, head turned to the right, and secured in a three-pin cranial fixation device. A question mark-shaped incision and musculocutaneous flap were made, beginning just above the zygomatic arch, curving superior and posterior to the ear, and extending to the anterior hairline along the superior temporal line ( Fig. 2 ). A temporal craniotomy was performed, and the dura mater was opened in a double-Y fashion. Resection of the neocortex and mesial temporal structures was performed through a completely subpial technique. Dynamic retraction using a brain spatula was employed during resection of the mesial temporal structures. The amygdala was resected using bipolar coagulation and suction, followed by en bloc hippocampectomy, with the inferior choroidal point serving as the posterior limit of resection. Bipolar coagulation was routinely used during dissection and hemostasis at a setting of 7, with continuous irrigation using normal saline provided by the assistant during coagulation. During the final inspection using a brain spatula near the inferior choroidal point, a transient decrease in motor-evoked potentials from the ipsilateral hand and foot was observed, which partially recovered before completion of the procedure.

**Fig. 2:**
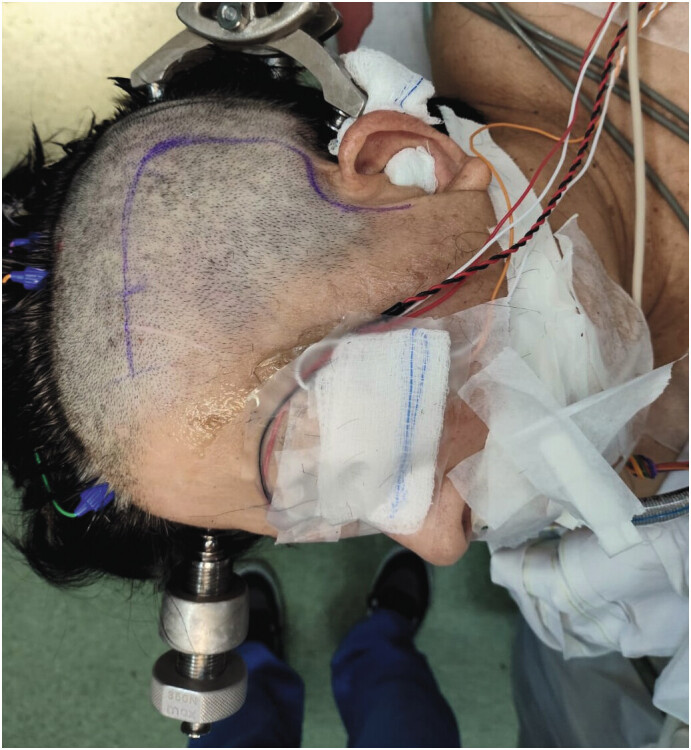
Positioning of the patient, showing the curvilinear skin incision along the superior temporal line reaching just anterior to the tragus and at the upper border of the zygomatic arch.

There was no significant bleeding during the procedure. The procedure was closed routinely, and a subgaleal drain was left in place for 48 h. The patient was extubated and transferred to the intensive care unit and then to the regular ward. Muscle strength was grade 5/5 in all four extremities, and no facial paralysis was initially evident. Immediate postoperative brain computed tomography showed a good extent of resection of temporal lobe neocortex and mesial structures without hematomas in the surgical bed ( Fig. 3 ).

**Fig. 3:**
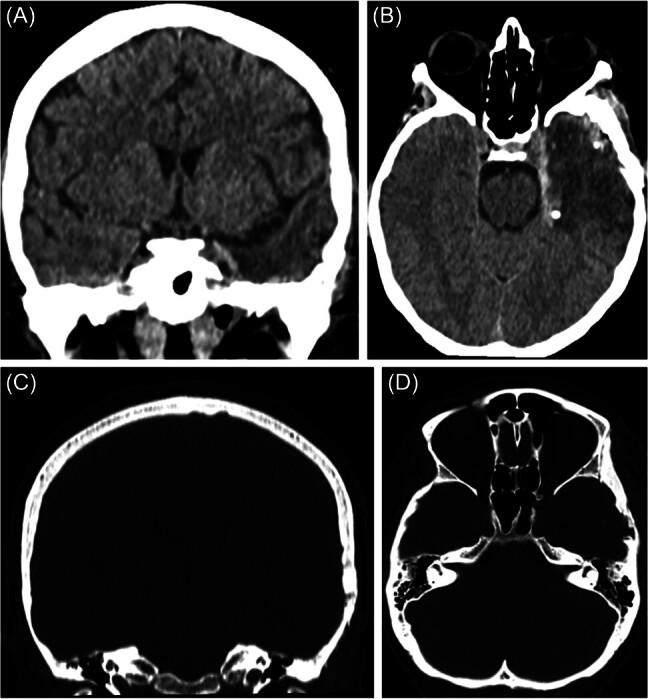
Postoperative brain CT. Coronal and axial parenchymal window views showing no evidence of intracranial hematoma and a good extent of resection ( **A, B** ). Coronal and axial bone window views showing skull base fractures or bony dehiscence over the facial hiatus ( **C, D** ). CT, computed tomography.

## Outcome

The patient was discharged 72 h later and returned for a follow-up visit a week later. A House–Brackmann grade 4 peripheral facial paralysis on the left side was noted, which the family member reported noticing the day after arriving home ( Fig. 4 ). The patient reported ear pain and hyperacusis on the left side. Muscle strength in all four extremities remained 5/5, with no evidence of blisters or crusting in the external auditory canal. Postoperative brain computed tomography (CT) scan shows no evidence of intracranial hematomas or bony defects on the petrous bone. Oral corticosteroids were started with progressive tapering over the next 10 days. Two weeks after starting corticosteroids, the patient showed evident improvement in the left peripheral facial paralysis ( Fig. 4 ).

**Fig. 4:**
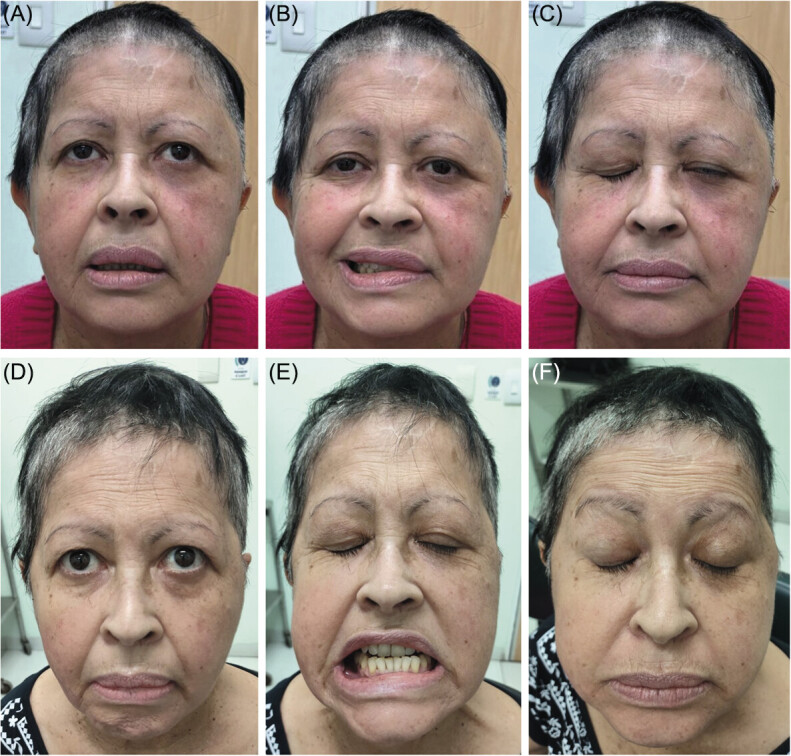
One-week postoperative photos evidencing peripheral facial paralysis HB4 ( **A–C** ). Three-week postoperative photos showing evidence of almost complete resolution of the facial paralysis ( **D–F** ). HB4, House–Brackmann grade 4.

## Discussion

Despite the decrease in use over the years, due to the introduction of new and better antiseizure drugs, ATL is the most performed surgery for the treatment of refractory epilepsy, accounting for 59 to 74% of cases. 1 2

In general, complications of ATL occur in 15 to 17% of cases, the most frequent being psychiatric disorders, visual field deficit, cognitive disorders, hemiparesis/hemiplegia, language deficit, infections, hemorrhages, cranial nerve deficits, hydrocephalus, extra-axial collections, and medical complications. 7 8 9

Cranial nerve deficits occur in approximately 3% of ATLs. 9 The most affected cranial nerves are the third and fourth cranial nerves, being injured in the resection of anterior and posterior mesial structures, respectively. 9

Surgery for resection of vestibular schwannoma and microvascular decompression for hemifacial spasm are the most common neurosurgical procedures in which delayed facial paralysis can occur. 10 11

Peripheral nerve palsy can occur because of idiopathic causes, infection, tumor, trauma, pregnancy, and autoimmune issues. 12 The House–Brackmann classification is one of the most widely used to characterize the severity of peripheral facial paralysis. 13 Idiopathic Bell’s palsy (IBP) is the most common type of peripheral facial paralysis and is most strongly associated with the reactivation of herpes simplex virus type 1, although other viral agents, including varicella zoster virus and human herpesvirus 6, are also commonly implicated. 14 The condition typically presents with paralysis of the facial muscles on the same side as the affected nerve, reflecting the loss of voluntary motor control. In some cases, additional manifestations may appear, such as pain in or around the ear, hyperacusis, and altered taste perception in the anterior tongue. 12 Despite these symptoms, approximately 70% of patients experience complete recovery of facial function. 12 The American Academy of Otolaryngology-Head and Neck Surgery recommends the use of oral steroids, preferably within 72 h of onset of symptoms, with or without the concomitant use of antiviral agents, to improve facial nerve recovery in IBP. 14

In the case series published by Anderson et al in 1991, delayed peripheral facial paralysis occurred in 4/110 (3.6%) of temporal lobectomies performed for the treatment of epilepsy. 4 After ruling out damage to the extratemporal facial nerve during the approach, Anderson and his group proposed that the most common explanation is facial nerve damage near the geniculate ganglion, caused by either heat or mechanical trauma occurring during the resection of mesial temporal lobe structures in the three ipsilateral cases but not in the remaining contralateral case, which was treated as IBP. 4 In the 1960s, Rhoton et al examined 100 temporal bones, and in 15% of them, they found the absence of bone over the geniculate ganglion, making it more susceptible to trauma during surgical procedures. 15 It is known that direct manipulation of the facial nerve and immunosuppression due to surgical stress can reactivate viruses of the Herpesviridae family that may be lodged in the geniculate ganglion, mainly herpes virus type 1, herpes virus type 2, and varicella zoster virus. 16 Facial nerve lesions could also occur at the level of the extratemporal trunk of the facial nerve if the skin incision is placed approximately 26 mm below the zygomatic arch at the level of the tragus, or at the level of the frontotemporal branch if placed 18.6 mm anterior to the tragus. 17 The use of local anesthetics for either dental procedures or scalp blocks can produce peripheral facial paralysis as a side effect due to direct needle injury, chemical injury, or compression due to hematoma. 18 19 Although cases of facial paralysis secondary to skull fracture caused by cranial fixation pins were not found in the literature, this complication should nevertheless be considered, as skull fractures associated with cranial fixation devices have been reported in up to 2.4% of cases. 20

Although a definitive etiological mechanism cannot be established in the presented case, the temporal relationship, clinical features, exclusion of alternative causes, and favorable response to corticosteroids suggest delayed peripheral facial nerve palsy due to viral reactivation associated with surgery.

## Conclusion

Delayed peripheral facial paralysis is an uncommon but clinically significant complication following ATL. Although rare, its occurrence underscores the need for neurosurgeons to remain vigilant in the postoperative period, even when no immediate cranial nerve deficits are observed. The case presented highlights the potential roles of viral reactivation and surgical trauma as underlying mechanisms, emphasizing the importance of timely recognition and corticosteroid therapy to optimize recovery. Increasing awareness of this rare complication can improve early diagnosis and guide appropriate treatment.
